# Investigation of stenotic jets using 3D-PC-UTE

**DOI:** 10.1186/1532-429X-14-S1-W55

**Published:** 2012-02-01

**Authors:** Karin Markenroth Bloch, Anders Nilsson, Freddy Ståhlberg

**Affiliations:** 1 Clinical Science, Philips Healthcare, Lund, Sweden; 2 Dept. of Medical Radiation Physics, Lund University, Lund, Sweden; 3 Dept. of Diagnostic Radiology, Lund University, Lund, Sweden

## Background

Conventional phase contrast (PC) velocity measurements fail in situation of high velocities and complex flow, as after a stenosis. Specifically, volume flow and peak velocities are underestimated, and velocity-to-noise ratio (VNR) is decreased due to flow voids. These effects are much dependent on the echo time (TE). Reducing TE will drastically improve the results, both concerning VNR and accuracy.

The method of ultra-short TE (UTE) allows for sub-millisecond TE, and has recently been implemented for 2D through-plane PC(2D PC-UTE). This work aims to investigate peak velocities and visualize stenotic jets in a 3D volume using PC-UTE.

## Methods

Two flow phantoms, PH1 and PH2, were used in two separate scanning sessions. PH1 consisted of plastic tubes, d=20 mm, in which were placed discs with central, circular holes of d=3 and 9 mm (s1 and s2). PH2 is a commercially available phantom mimicking a carotid bifurcation with a 60% stenosis in the left branch. A steady flow of water doped with Gadovist was pumped through the phantoms. In PH1, the 3D volume covered both s1 and s2. In PH2, the volume covered one slice before the stenosis. Two 3D PC sequences were used: a conventional, cartesian 3D PC (3D PC), and a radial 3D PC-UTE. Both had 12 slices, 14 time frames, TR=6 resp. 8 ms, ETL=5, voxel size 1.5x1.5x3 mm^3^ and through-plane velocity encoding, v_enc_=700 cm/s. The important difference is TE, which is 3.7 ms for the 3D PC and 0.76 ms for 3D PC-UTE.

For analysis, ROI’s were drawn over the vessel of interest. In PH1, the ROI was chosen with a diameter of 20 mm, and identical ROIs were used for all slices. For PH2, the ROI’s were drawn manually on the velocity images. In both phantoms, identical ROIs were used in the analysis of both datasets.

A quantitative analysis of the peak velocities was made by extracting the peak velocity in each ROI, and averaging that over the time frames for each slice. The standard deviation of the peak velocity was also calculated using data from the different time frames.

## Results

**Figure 1:**
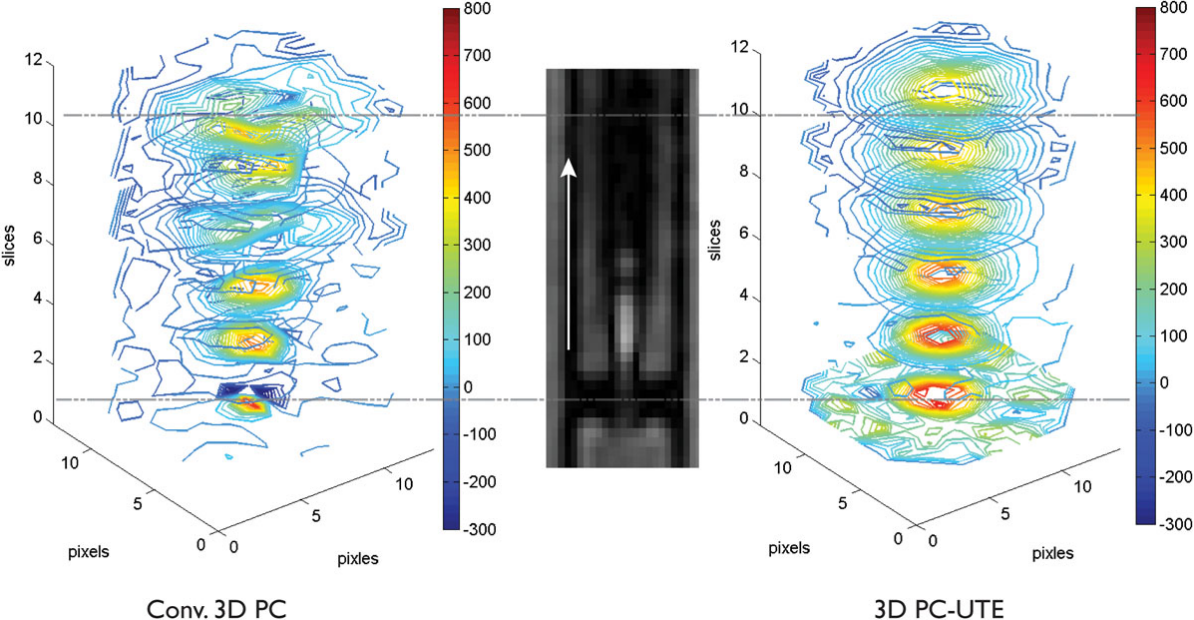
Contour plots of the through-plane velocity in the jet after the 3 mm stenosis. The 3D PC-UTE sequence has less artefacts and measures a higher peak velocity than the conventional sequence.

**Figure 2:**
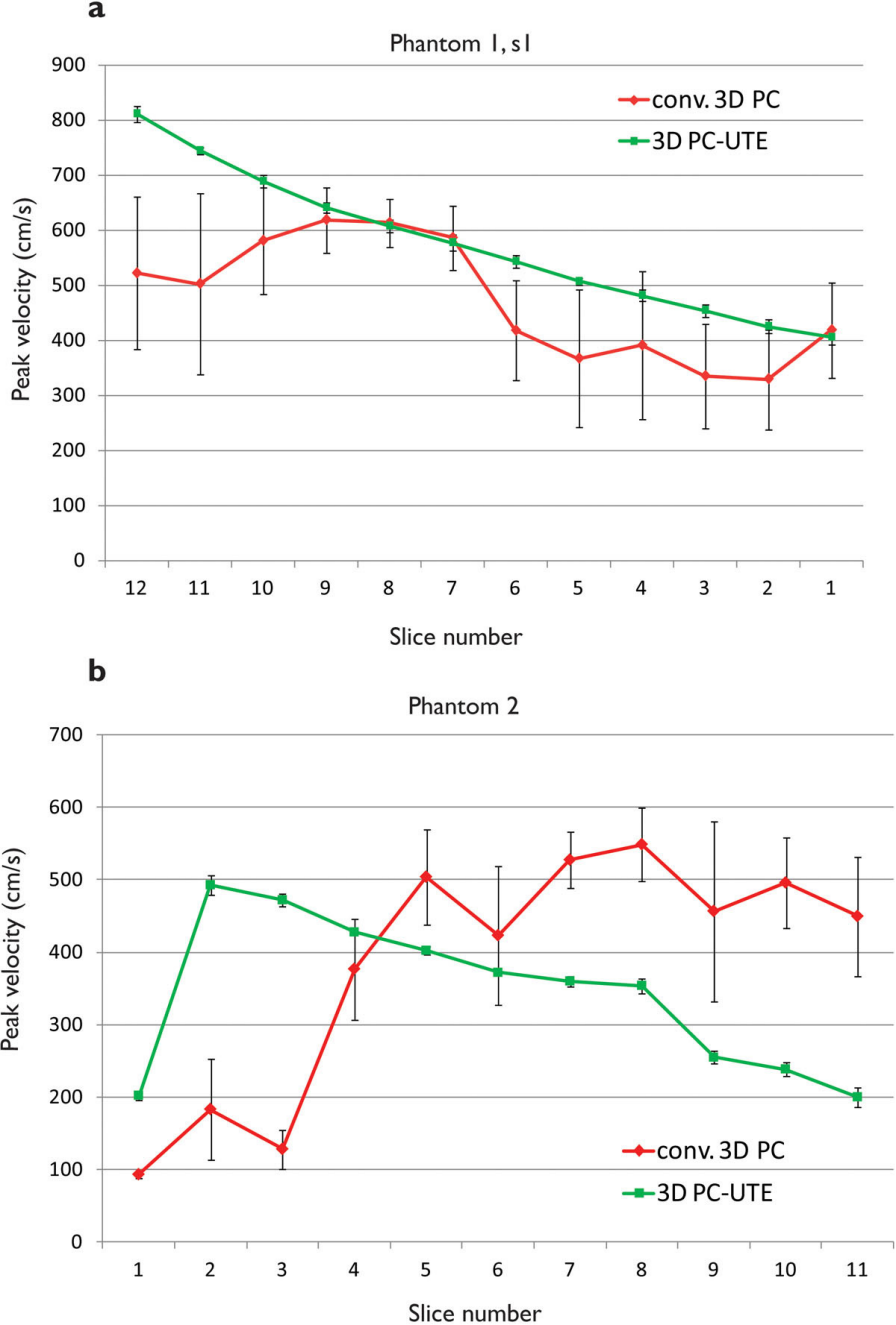
The figure shows the measured peak velocity in the vessel for each slice. The measure is an average over the time frames, and the error bars represent one standard deviation. Panel (a) shows the data for the smaller stenosis, s1, in phantom PH1. The conventional sequence (red diamonds) shows much higher standard deviations, and lower peak velocity than the 3D PC-UTE (green squares). Panel (b) shows the corresponding data for phantom 2. Again, the conventional sequence (red diamonds) shows much larger standard deviations than the 3D PC-UTE data (green squares). From the 3D PC-UTE data, the peak velocity is seen in the slice in the stenosis, after which the velocity drops. In the conventional data, the peak velocity is given to occur further downstream and keep more or less constant, which is not physically realistic.

In Figure 1, artefacts are evident in the 3D PC contour plots, while the 3D PC-UTE shows a more concentric jet. Figure 2 shows the peak velocities for (a) s1 in PH1 and (b) PH2. In the 3D PC-UTE data, the peak velocity occurs in the slice closest to the stenosis and then decreases in slices more distal to the obstruction. In 3D PC data sets, peak velocity has a much larger variation, and the absolute peak occurs in more distal slices. A limitation of the study is that true peak velocity is now know, but estimations based on the known flow rate and measured jet areas indicate that the true value is at least as high as the 3D PC-UTE estimate.

## Conclusions

3D PC-UTE is shown to improve the accuracy and reduce variance in measurements of peak velocity in stenotic jets. The 3D implementation allows for finding the peak velocity even if it is unknown a priori where in the jet it occurs or if there is through-plane motion during the cardiac cycle.

## Funding

The Swedish Strategic Foundation.

